# Supplementary material for: Metaplastic Carcinoma of the Breast: Case Series of a Single Institute and Review of the Literature
Source: Med Sci (Basel). 2023 May 19;11(2):35. doi: 10.3390/medsci11020035 (PMC10204473; doi:10.3390/medsci11020035)
Supplement: Supplementary file 1 [file medsci-11-00035-s001.zip › medsci-2342987-supplementary.pdf]

Review

Metaplastic Carcinoma of the Breast: Case Series of a Single Institute and Review of the Literature

Alkistis Papatheodoridi, Eleni Papamattheou, Spyridon Marinopoulos, Ioannis Ntanasis-Stathopoulos, Constantine Dimitrakakis, Aris Giannos, Maria Kaparelou, Michalis Liontos, Meletios-Athanasios Dimopoulos and Flora Zagouri

Table S1. Baseline characteristics of Alexandra Hospital patients.

	Age	Stage at diagnosis	ER	PR	Her2	Ki67	Surgery	Chemotherapy	Radiotherapy	Histological type
Pt1	71	IIB	-	-	-	High	YES	YES	YES	squamous
Pt2	72	IIA	-	-	-	Low	YES	YES	YES	matrix producing
Pt3	68	IIA	-	-	-	High	YES	YES	YES	mixed
Pt4	81	IIA	-	-	-	High	YES	NO	NO	squamous
Pt5	48	IIA	+	-	-	High	YES	YES	NO	spindle cell carcinoma
Pt6	72	IIA	-	-	-	High	YES	YES	YES	mixed
Pt7	58	IIB	-	-	-	High	YES	YES	YES	spindle cell carcinoma
Pt8	54	IIA	-	-	-	High	YES	YES	YES	mixed
Pt9	71	IA	-	+	-	High	YES	YES	NO	mixed
Pt10	41	IIA	+	+	-	High	YES	YES	YES	mixed
Pt11	32	IIB	-	-	-	High	YES	YES	YES	squamous
Pt12	75	IIB	-	-	-	High	YES	YES	YES	squamous
Pt13	59	IIA	-	-	-	High	YES	YES	YES	mixed
Pt14	73	IIIC	-	-	-		YES	YES	NO	mixed
pt15	58	IIA	-	-	-		YES	YES	YES	mixed
pt16	36	IIB	-	-	-		YES	YES	YES	mixed
pt17	49	IIA	-	-	-		YES	YES	NO	matrix producing
pt18	42	IIB	-	-	-		YES	YES	YES	mixed
pt19	83	IIA	-	-	-	High	YES	NO	NO	matrix producing
pt20	58	IIB	-	-	-	High	YES	YES	NR	matrix producing
pt21	35	IIB	-	-	-	High	YES	NO	NO	mixed
pt22	41	IIA	-	-	-	low	YES	YES	YES	squamous
pt23	62	IIA	-	-	-	High	YES	YES	YES	Metaplastic
pt24	64	IIA	-	-	+	low	YES	YES	YES	mixed
pt25	82	IV	-	+	-	low	YES	NO	NO	Metaplastic
pt26	55	IA	-	-	-	High	YES	YES	YES	Metaplastic with osseous differetion
pt27	85	IIA	-	-	-		YES	NO	NO	mixed
pt28	59	IIA	-	-	-	low	YES	YES	YES	mixed
pt29	80	IA	-	-	-		YES	YES	YES	Metaplastic
pt30	45	IA	-	-	-		YES	YES	YES	squamous
pt31	58	IA	-	-	-	high	YES	YES	YES	Metaplastic
pt32	78	IIIB	+	-	-		YES	YES	YES	Metaplastic
pt33	70	IIIA	-	-	-	low	YES	YES	NO	Metaplastic with chondrous & osseous differetion
pt34	65	IA	-	-	-		YES	YES	YES	Metaplastic
pt35	65	IIB	-	-	-		YES	YES	NO	mixed
pt36	55	IIA	-	-	-		YES	YES	NO	squamous
pt37	66	IIA	-	-	-	high	YES	YES	YES	mixed
pt38	56	IIA	-	-	-		YES	YES	NO	mixed
pt39	70	IIIA	-	-	-		YES	YES	YES	mixed
pt40	68	IA	-	-	-		YES	YES	YES	squamous
pt41	60	IIA	-	-	-	low	YES	YES	YES	mixed
pt42	43	IIA	-	-	-	low	YES	YES	YES	squamous
pt43	77	IIB	-	-	-	high	YES	YES	YES	Metaplastic with chondrous differetion
pt44	65	IIA	+	+	+	low	YES	YES	YES	mixed
pt45	82	IA	-	-	-	low	YES	NO	NO	mixed
pt46	88	IIA	-	-	-	low	YES	YES	NO	squamous
